# Effects of Transcranial Direct Current Stimulation Targeting Dorsolateral Prefrontal Cortex and Orbitofrontal Cortex on Somatic Symptoms in Patients With Major Depressive Disorder: A Randomized, Double‐Blind, Controlled Clinical Trial

**DOI:** 10.1111/cns.70110

**Published:** 2024-11-08

**Authors:** Shuxiang Shi, Haijing Huang, Mengke Zhang, Yiming Chen, Weichieh Yang, Fan Wang, Shuqi Kong, Ni Zhou, Zheyi Wei, Shentse Chen, Dongbin Lyu, Chenglin Wu, Qinte Huang, Qinting Zhang, Wu Hong

**Affiliations:** ^1^ Shanghai Mental Health Center Shanghai Jiao Tong University School of Medicine Shanghai China; ^2^ Shanghai Key Laboratory of Psychotic Disorders Shanghai China; ^3^ Mental Health Branch, China Hospital Development Institute Shanghai Jiao Tong University Shanghai China; ^4^ Shanghai Hongkou Mental Health Center Shanghai China; ^5^ Shanghai Pudong New Area Mental Health Center Shanghai China; ^6^ Shanghai Key Lab of Forensic Medicine, Key Lab of Forensic Science, Ministry of Justice, Shanghai Forensic Service Platform Academy of Forensic Science Shanghai China

**Keywords:** major depressive disorder, somatic anxiety, somatic symptoms, transcranial direct current stimulation

## Abstract

**Aim:**

There is a lack of research on transcranial direct current stimulation (tDCS) for the treatment of somatic symptoms in major depressive disorder (MDD) and the suitable stimulating brain region. We investigated the efficacy of tDCS targeting the dorsolateral prefrontal cortex (DLPFC) versus orbitofrontal cortex (OFC) on depressive somatic symptoms and somatic anxiety in patients with MDD and aimed to identify the appropriate stimulating brain regions.

**Methods:**

In this randomized, double‐blind, sham‐controlled study, a total of 70 patients diagnosed with MDD were randomly allocated into DLPFC group, OFC group, and Sham group. Subjects participated in 2 weeks of 10 primary interventions and subsequently 2‐week maintenance interventions weekly (20 min, 2 mA).

**Results:**

The DLPFC group showed a more significant improvement in somatic symptoms compared to the Sham group at week 2. At the maintenance and follow‐up stages, the DLPFC group outperformed the Sham and OFC groups, but the difference with the Sham group was not significant. Neither active group demonstrated superiority over the Sham group in improving depression and anxiety.

**Conclusion:**

In conclusion, the tDCS targeting DLPFC may be a potentially effective therapeutic target for alleviating somatic symptoms in patients with MDD.

## Graphical abstract

Somatic symptoms are common manifestations in MDD patients. However, there is a lack of research on tDCS for treating somatic symptoms in MDD. This study found tDCS targeting DLPFC significantly improved somatic symptoms in MDD patients, suggesting it an effective target of tDCS for somatic symptoms in MDD.

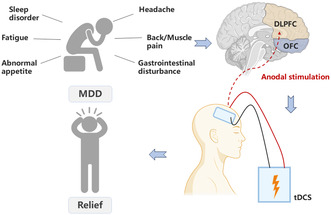

## 1 Introduction

It is estimated that approximately 3.8% of the global population, equating to around 280 million individuals, are afflicted with major depressive disorder (MDD) [1]. The Global Burden of Disease Study identifies MDD as a leading contributor to worldwide disease burden, underscoring the urgency of effective treatment strategies [2].

Patients diagnosed with MDD frequently exhibit somatic symptoms, which are primarily categorized into vegetative, pain, and non‐pain symptoms. Vegetative symptoms encompass sleep disturbances, changes in appetite, and lack of energy. Pain symptoms include headache, back pain, gastrointestinal disturbances, and musculoskeletal pain. Non‐pain symptoms comprise dizziness, palpitations, dyspnea, and shortness of breath [3, 4, 5]. Moreover, somatic anxiety is prevalent among MDD patients and is linked to the response to antidepressant treatment [6]. An international multicenter study revealed that approximately two‐thirds of MDD patients exhibit somatic symptoms [7]. Furthermore, these somatic symptoms often coexist with depression and anxiety [8]. The concurrent presence of somatic symptoms and mental health issues amplifies the disease burden by impacting health outcomes, functionality, and economic costs [9, 10, 11]. Antidepressants are the most frequently prescribed medication for treating somatic symptoms. However, these symptoms have shown less responsiveness to conventional antidepressant treatment compared to depressive symptoms [12, 13]. Therefore, it is imperative to explore effective interventions to address somatic symptoms in MDD patients.

In recent years, innovative noninvasive transcranial brain stimulation techniques such as repetitive transcranial magnetic stimulation (rTMS) and transcranial direct current stimulation (tDCS) have been advanced for depression treatment [14, 15, 16]. Compared to rTMS, tDCS treatments are more cost‐effective and can be implemented in diverse environments, including home‐based care [17]. The tDCS technique injects a weak direct current through scalp electrodes, and the current passes through several anatomical layers to the brain, where it modulates neuronal excitability and cortical activity via changing the membrane potential according to stimulation parameters [18, 19]. Meta‐analysis based primarily on studies of patients with combination therapy, with or without medication, and with unipolar or bipolar depression has shown that tDCS can yield low to moderate antidepressant effects [20, 21]. Meanwhile, a recent meta‐analysis revealed that the impact of tDCS monotherapy was substantial but exhibited considerable heterogeneity [22]. It has also been suggested that active tDCS is not superior to sham tDCS in the treatment of unipolar or bipolar depression [23]. Although tDCS is one of the promising treatments for MDD according to evidence‐based guidelines, the optimal protocol is still worth exploring [24].

Studies of tDCS treating MDD typically position anodes on the hypoactive left dorsolateral prefrontal cortex (DLPFC). This positioning aims to enhance local activity and restore normal function [25]. Evidence indicates that tDCS treatment, when targeting the DLPFC, is not only effective in addressing acute depressive episodes but also beneficial in managing the follow‐up of depression, suggesting that tDCS intervention may augment the benefits derived from the acute treatment phase [26]. Cathode placement on the right DLPFC or right supraorbital or frontotemporal regions results in different current distributions, which explains the various outcomes of different stimulation protocols [27]. Due to the imbalance of left–right DLPFC in MDD characterized in neuropsychological regard, research found that one rationale for placing electrodes over DLPFC in depression is improving dysfunctional cognition underlying negative mood in depression [28, 29]. The orbitofrontal cortex (OFC) has been consistently involved in neural systems important for emotion regulation and stress response, as well as in neural markers of outcome and treatment response in depression [30]. A review of OFC corticostriatal circuits suggests that the OFC circuitry could be a promising target for therapeutic brain stimulation in psychiatric disorders [31]. Furthermore, research has found that rTMS treatment using inhibitory stimulation of the right OFC improves depressive symptoms in patients with MDD comorbid with obsessive–compulsive disorder [32, 33]. Nonetheless, there is a paucity of studies investigating cathode‐targeted OFC‐tDCS for depression. Therefore, it is necessary to further investigate the efficacy of tDCS with different targets in treating depression.

In addition to regulating the mood of MDD patients, tDCS can also treat pain symptoms, which are one of the core symptoms of somatic symptoms [34]. The DLPFC plays a crucial role in pain management by modulating both cortico‐subcortical and cortico‐cortical pathways, involving the engagement of both somatosensory regions and regions responsible for processing emotionally significant stimuli [35]. The most commonly used tDCS treatment paradigm for pain control is anodal stimulation of the left DLPFC [36]. Additionally, tDCS protocols involving anodal stimulation of the DLPFC have proven beneficial in treating insomnia and sleep disturbances in neuropsychiatric populations [37]. Research has also revealed a correlation between the OFC and somatic symptoms. Specifically, more somatic symptoms are associated with less surface area of left OFC [38]. Similarly, a neuroimaging study showed that the higher the connectivity strength of OFC, the greater the improvement of somatic symptoms, and considered that OFC played an important role in the cognitive process of pain perception [39]. There has also been a case report finding that tDCS incorporating anodic stimulation of the left DLPFC and cathodic stimulation of the right OFC can improve somatic anxiety [40]. Consequently, modulation of DLPFC and OFC could potentially alleviate somatic symptoms, in alignment with their roles in cognitive and emotional regulation. However, there is still a lack of evidence for the effectiveness of tDCS targeting DLPFC and OFC in the treatment of somatic symptoms in MDD patients, which warrants further investigation.

In summary, the therapeutic efficacy of tDCS targeting various regions in alleviating somatic symptoms in MDD patients remains ambiguous, and relevant clinical studies are lacking. Consequently, we initiated a double‐blind, randomized, sham‐controlled trial with the objective of comparing the efficacy of tDCS targeting DLPFC and OFC on somatic symptoms in MDD.

## 2 Methods

### 2.1 Study Design

A randomized, double‐blind, sham‐controlled design was used in this study. The trial was registered at the Chinese Clinical Trial Registry (ChiCTR2000034671). All participants provided written informed consent. All evaluators and tDCS therapists received consistency training in advance. According to the random sequence table prepared by the third party, participants were assigned to three research groups (OFC group, DLPFC group, and Sham group). We set up the three conditions using the built‐in double‐blind program of the device (double‐blind “study mode”). The stimulation parameters for the sham group were the same as for the active group.

### 2.2 Participants

#### 2.2.1 Inclusion and Exclusion Criteria

Right‐handed patients were recruited from the outpatient clinic of Shanghai Mental Health Center from August 2020 to November 2021. Inclusion criteria were as follows: (a) patients diagnosed with major depressive episode according to the Diagnostic and Statistical Manual of Mental Disorders 5th (DSM‐5) criteria and established using the Mini‐International Neuropsychiatric Interview (MINI; Version 5.0.0); (b) the total score of 17‐item Hamilton Depression Rating Scale (HAMD‐17) ≥ 14 and the score for item 1 of the HAMD‐17 ≥ 2; (c) the total score of Depression and Somatic Symptoms Scale (DSSS) ≥ 3 and the score for somatic subscale (SS) ≥ 1; (d) aged 18–65 years old; and (e) possessing the ability of listening, speaking, reading, writing, and comprehension to complete the study.

Exclusion criteria were as follows: high suicide risk as defined by psychiatrists; other psychiatric and neurological disorders; metal implants in the head; substance abuse or dependence; current treatment with benzodiazepines (as this may alter the effects of tDCS); mania and hypomania episode defined as a score < 5 on the Young Manic Rating Scale (YMRS); pregnancy; had received other neuromodulation therapy within the last 3 months.

Throughout the intervention period, participants either did not receive medication or maintained a stable antidepressant regimen and dosage, which did not change for at least 2 weeks before being included in this study.

#### 2.2.2 Sample Size

The sample size for this pilot study was estimated based on previous studies [41, 42]. One‐way analysis of variance (ANOVA) (PASS 15) was used to calculate the sample size. As a pilot study, the parameters were set as equal numbers of participants in the three groups, effect size *f* = 0.50, *α* = 0.05, power = 80%, and a dropout rate of 20%. Sample sizes of 18, 18, and 18 were obtained for each study group and 54 for the total.

### 2.3 tDCS Protocol

The Starstim Multichannel stimulator (Neuroelectrics, Spain) was used to perform tDCS stimulation. Sponge electrodes (5 × 5 cm) were placed over the OFC and DLPFC areas at Fp1/Fp2 or F3/F4, respectively, according to the international system of 10–20 EEG [43]. The anode was placed over F3 (left) and the cathode over F4 (right) in the DLPFC group. The anode was placed over Fp1 (left) and the cathode over Fp2 (right) in the OFC group. All three groups included a 30s ramp‐up and a 30s ramp‐down, but during the 20‐min stimulation period, the active stimulation was 2 mA and the sham stimulation was 0 mA.

### 2.4 Study Procedure

Participants were scheduled to complete 10 interventions during the initial 2‐week period and 1 intervention per week for the following 2 weeks based on a previous randomized controlled trial [44]. All baseline (week 0) assessments were completed prior to the initial intervention. Clinical scale assessments were conducted at the end of the 10 interventions (week 2) and at the end of maintenance treatment (week 4), and follow‐up assessments were conducted at weeks 6 and 8 (compared with baseline).

### 2.5 Measures of Outcomes

The severity of depression was assessed with HAMD‐17 [45] and Maier subscale (MS), which focuses on core depression symptoms and excludes somatic symptoms [46]. DSSS is a simple and self‐administered scale with 22 items, and it has been demonstrated to have good reliability and validity for depression, with a high correlation with HAMD outcomes [47]. Compared to the conventional scales, the DSSS evaluates somatic symptoms more accurately [47, 48]. DSSS items were grouped into two domains: depression subscale (DS) and SS. Depressive and somatic symptoms were assessed with the DSSS score and SS score. The Hamilton Anxiety Rating Scale (HAMA) was used to quantify anxiety severity [49]. HAMA is a 14‐item measure consisting of psychic anxiety (PA) and somatic anxiety (SA). In this study, HAMA, PA, and SA scores were used to evaluate the anxiety and somatic symptoms of anxiety.

The primary outcomes were the reduction rate of DSSS, SS and SA score from baseline to week 2 and 4. Secondary outcomes were the reduction rate of HAMD‐17, MS, HAMA, and PA score at week 2 and 4. Other outcomes included the assessment of each scale during the follow‐up period and predictive analysis.

### 2.6 Adverse Effects and Safety

Adverse effects were assessed using the tDCS Adverse Effects Questionnaire, with severity ratings ranging from 0 (none) to 3 (severe) [50]. The YMRS [51] was used to detect any possible hypomanic and manic episodes. Suicidal ideation and behaviors during the intervention period were also evaluated by experienced psychiatrists.

### 2.7 Statistical Analysis

Statistical analysis was performed using SPSS26.0 software (IBM). All statistical results were two‐sided tests, and *p* < 0.05 was considered statistically significant. All data were tested for normality by Shapiro–Wilk test. GraphPad Prism 9.0.0 version was used to graph.

Differences in participant characteristics among three groups were tested using *χ* ^2^ tests for categorical variables and one‐way ANOVA for continuous variables. The efficacy results of the three intervention groups were analyzed by one‐way ANOVA or Welch test and differences between groups were analyzed using Bonferroni or Games–Howell post hoc test, which depended on homogeneity of variance. Non‐normally distributed continuous variables were compared using a nonparametric test. No data imputation was performed. The changes of scores over time in different tDCS intervention groups at each time point and the interaction effects were compared by repeated‐measures ANOVA with Bonferroni post hoc test.

The general linear model and generalized linear model were used to investigate whether any predictor factors, including clinical characteristics (age at first onset, use of medication, baseline score) or demographic measures (gender, age, education, marital status, employment status), influenced tDCS outcomes. These models included factors of the group, the predictor factor, and the interaction between these factors. The correlation between the reduction rate and these predictor factors was analyzed by Pearson's correlation analysis and Spearman's correlation analysis.

## 3 Results

### 3.1 Clinical and Demographic Characteristics

A total of 155 patients met the inclusion criteria, of which 85 were excluded for various reasons, ultimately leaving 70 patients who were randomly assigned to the three groups to participate in the study. As shown in Table S1, 57 (81.4%) and 52 (74.3%) participants completed the 2‐week and 4‐week interventions and assessments, respectively. The numbers of individuals who completed the 6‐week and 8‐week follow‐ups were 41 (58.6%) and 27 (38.6%). Reasons for dropping out were similar among three groups (Figure S1).

Demographics and baseline clinical characteristics of patients are listed in Table 1. Except for the score of QIDS‐16SR (*F*(2,67) = 3.498, *p* = 0.036), there were no statistically significant differences among the three groups at baseline.

**TABLE 1:** Comparison of demographic and clinical characteristics among three groups at baseline.

	OFC group (*n* = 23)	DLPFC group (*n* = 23)	Sham group (*n* = 24)	*F*/*χ* ^2^	*p*
	Mean (SD)/*n* (%)				
Gender (male)	6 (26.1%)	8 (34.8%)	4 (16.7%)	2.020	0.364
Age (years)	26.7 (8.5)	25.7 (5.4)	27.3 (5.6)	0.326	0.723
Education (years)	14.7 (3.1)	16.0 (4.0)	15.6 (2.6)	0.940	0.396
Marital status					
Single	16 (69.6%)	15 (65.2%)	15 (62.5%)	4.905	0.768
In relationship	2 (8.7%)	5 (21.7%)	5 (20.9%)		
Married	4 (17.4%)	3 (13.0%)	4 (16.7%)		
Divorced	1 (4.3%)	0 (0.0%)	0 (0.0%)		
Work status					
Employment	11 (47.8%)	10 (43.5%)	10 (41.7%)	5.932	0.821
Student	6 (26.1%)	7 (30.4%)	6 (25.0%)		
Unemployment	6 (26.1%)	6 (26.1%)	8 (33.3%)		
Smoker	7 (30.4%)	2 (8.7%)	6 (25.0%)	3.505	0.173
Age at first onset	21.9 (7.0)	20.9 (5.6)	21.3 (6.7)	0.139	0.870
On medication	14 (60.9%)	7 (30.4%)	10 (44.3%)	4.419	0.110
DSSS	31.2 (11.9)	25.3 (11.2)	28.8 (10.3)	1.659	0.198
DS	20.2 (7.0)	17.7 (7.1)	21.0 (6.2)	1.513	0.228
SS	11.0 (6.1)	7.6 (5.5)	7.8 (6.5)	2.320	0.106
HAMD‐17	21.7 (3.8)	19.8 (3.4)	21.3 (3.1)	1.971	0.147
MS	10.74 (1.6)	10.35 (2.2)	10.75 (1.8)	0.505	0.777
MADRS	27.1 (8.5)	25.4 (7.7)	30.5 (7.8)	2.553	0.085
QIDS‐SR16	21.3 (5.7)	17.0 (5.8)	18.6 (4.9)	3.498	0.036*
SSI	15.7 (13.9)	15.4 (12.9)	18.0 (14.2)	0.257	0.774
HAMA	16.3 (4.6)	14.0 (5.6)	15.0 (5.5)	1.152	0.322
SA	5.2 (2.9)	4.1 (2.9)	3.8 (3.1)	1.423	0.248
PA	11.1 (2.5)	9.9 (3.7)	11.2 (3.5)	1.141	0.326

Abbreviations: DLPFC, dorsolateral prefrontal cortex; DS, depression subscale; DSSS, Depression and Somatic Symptoms Scale; HAMA, the Hamilton Anxiety Rating Scale; HAMD‐17, the 17‐item Hamilton Depression Rating Scale; MADRS, Montgomery–Asberg Depression Rating Scale; MS, Maier subscale; OFC, orbitofrontal cortex; PA, psychic anxiety; QIDS‐SR16, the 16‐Item Quick Inventory of Depressive Symptomatology; SA, somatic anxiety; SS, somatic subscale; SSI, Beck Scale for Suicide Ideation.

*p < 0.05.

### 3.2 Effect on Somatic Symptoms

The primary outcomes of tDCS are presented in Tables 2 and 3. The reduction rate of the DSSS score at week 2 after primary treatment was 35.71% ± 26.42% in the OFC group (*n* = 20), 52.54% ± 26.65% in the DLPFC group (*n* = 18), and 27.59% ± 17.45% in the Sham group (*n* = 19), showing a significant difference among three groups (*F*(2,54) = 5.224, *p* = 0.008). The reduction rate of the SS score was 35.34% ± 39.35% in the OFC group, 61.21% ± 40.55% in the DLPFC group, and 19.745% ± 42.27% in the Sham group, also showing a significant difference among three groups (*H* (df = 2) = 8.156, *p* = 0.017). In terms of the reduction rate of the SA score, there was no significant difference among the three groups (*H* (df = 2) = 4.253, *p* = 0.119) (Table 2).

**TABLE 2:** DSSS, SS, and SA score and the reduction rate at week 2 after primary treatment.

	Group	Mean ± SD	Reduction rate (% ± SD)^a^	*F*/*H*	*p* ^b^
DSSS	OFC (*n* = 20)	20.90 ± 11.40	35.71 ± 26.42	5.224	0.008**
	DLPFC (*n* = 18)	12.67 ± 10.02	52.54 ± 26.65		
	Sham (*n* = 19)	19.74 ± 9.02	27.59 ± 17.45		
SS	OFC (*n* = 20)	7.85 ± 5.20	35.34 ± 39.35	8.156	0.017*
	DLPFC (*n* = 18)	3.33 ± 4.04	61.21 ± 40.55		
	Sham (*n* = 19)	5.05 ± 5.04	19.74 ± 42.27		
SA	OFC (*n* = 20)	2.45 ± 2.31	62.00 ± 36.13	4.253	0.119
	DLPFC (*n* = 18)	2.61 ± 2.00	17.47 ± 89.92		
	Sham (*n* = 19)	1.74 ± 2.18	31.88 ± 100.82		

Post hoc test				
	DSSS	Bonferroni adjusted *p* ^c^	SS	Bonferroni adjusted *p* ^c^
OFC vs. Sham		0.881		0.791
DLPFC vs. Sham		0.007**		0.014*
OFC vs. DLPFC		0.104		0.231

Abbreviations: DLPFC, dorsolateral prefrontal cortex; DSSS, Depression and Somatic Symptoms Scale; OFC, orbitofrontal cortex; SA, somatic anxiety; SS, somatic subscale.

^a^ Reduction rate, calculated as (score at baseline—score at week 2)/score at baseline) × 100.

^b^ *p* values of one‐way ANOVA (DSSS) or Kruskal–Wallis test (SS and SA).

^c^ Adjusted *p* values of Bonferroni post hoc test of the reduction rate.

*p < 0.05; **p < 0.01.

**TABLE 3:** DSSS, SS, and SA score and the reduction rate at week 4 after maintenance treatment.

	Group	Mean ± SD	Reduction rate (% ± SD)^a^	*p* ^b^
DSSS	OFC (*n* = 19)	18.11 ± 11.14	45.43 ± 27.22	0.012*
	DLPFC (*n* = 17)	7.71 ± 6.61	69.37 ± 21.92	
	Sham (*n* = 16)	13.06 ± 8.55	44.89 ± 38.88	
SS	OFC (*n* = 19)	5.79 ± 4.96	52.72 ± 40.43	0.009**
	DLPFC (*n* = 17)	1.00 ± 1.70	89.06 ± 18.59	
	Sham (*n* = 16)	3.19 ± 3.82	45.96 ± 72.07	
SA	OFC (*n* = 19)	3.84 ± 3.06	31.71 ± 46.00	0.170
	DLPFC (*n* = 17)	1.82 ± 1.98	45.10 ± 71.42	
	Sham (*n* = 16)	0.94 ± 1.29	37.50 ± 94.62	

Post hoc test				
	DSSS	Adjusted *p* ^c^	SS	Adjusted *p* ^c^
OFC vs. Sham		0.999		1.000
DLPFC vs. Sham		0.090		0.111
OFC vs. DLPFC		0.017*		0.008**

Abbreviations: DLPFC, dorsolateral prefrontal cortex; DSSS, Depression and Somatic Symptoms Scale; OFC, orbitofrontal cortex; SA, somatic anxiety; SS, somatic subscale.

^a^ Reduction rate, calculated as (score at baseline—score at week 4)/score at baseline) × 100.

^b^ *p* values of Welch (DSSS) or Kruskal–Wallis test (SS and SA).

^c^ Adjusted *p* values of Games–Howell (DSSS) and Bonferroni (SS and SA) post hoc test of the reduction rate.

*p < 0.05; **p < 0.01.

Post hoc analysis showed that at week 2, the reduction rate for DSSS was higher in the DLPFC group compared with the Sham group (adjusted *p* = 0.007), and the reduction rate for SS was also significantly different between DLPFC group and Sham group (adjusted *p* = 0.014) (Table 2).

After the maintenance treatment, the DSSS score at week 4 decreased 45.43% ± 27.22% in the OFC group (*n* = 19), 69.37% ± 21.92% in the DLPFC group (*n* = 17), and 44.89% ± 38.88% in the Sham group (*n* = 16). The reduction rate for SS was 52.72% ± 40.43% in the OFC group, 89.06% ± 18.59% in the DLPFC group, and 45.96% ± 72.07% in the Sham group. Significant differences were observed in the reduction rates for both DSSS (*F*(2,30.546) = 5.087, *p* = 0.012) and SS (*H* (df = 2) = 9.432, *p* = 0.009) among three groups at week 4. No significant difference was found among groups for the SA score reduction rates (*H* (df = 2) = 3.549, *p* = 0.170) (Table 3).

Post hoc multiple comparison at week 4 revealed that the DLPFC group outperformed the OFC group in both DSSS (adjusted *p* = 0.017) and SS (adjusted *p* = 0.008) score reduction rates, although there was no significant difference compared to the Sham group (Table 3).

**FIGURE 1:**
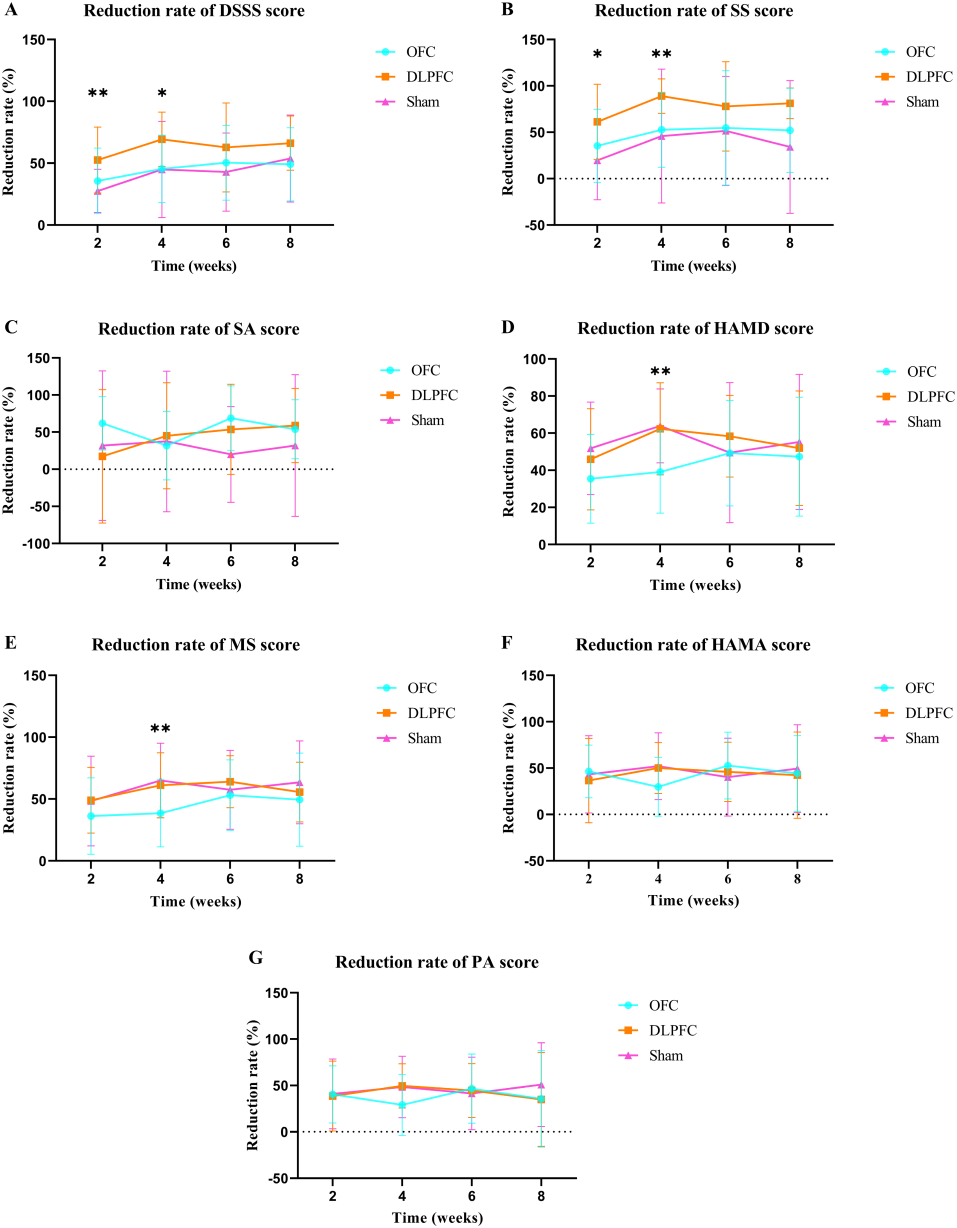
The reduction rate of scale score for three groups over intervention phases and follow‐up (mean ± SD). *p < 0.05, **p < 0.01. A: Line graph of DSSS reduction rate; B: Line graph of SS reduction rate; C: Line graph of SA reduction rate; D: Line graph of HAMD reduction rate; E: Line graph of MS reduction rate; F: Line graph of HAMA reduction rate; G: Line graph of PA reduction rate. DLPFC, dorsolateral prefrontal cortex; DSSS, Depression and Somatic Symptoms Scale; HAMA, the Hamilton Anxiety Rating Scale; HAMD‐17, the 17‐item Hamilton Depression Rating Scale; MS, Maier subscale; OFC, orbitofrontal cortex; PA, psychic anxiety; SA, somatic anxiety; SS, somatic subscale.

The reduction rate of the scale score for somatic symptoms is shown in Figure 1.

### 3.3 Effect on Depression and Anxiety

HAMD‐17 and MS scores were reduced in all three groups after the 2‐week and 4‐week interventions. However, there was no significant difference in the reduction rates at week 2 among three groups (HAMD‐17: *F*(2,54) = 2.120, *p* = 0.130; MS: *F*(2,54) = 1.010, *p* = 0.371) (Table 4).

**TABLE 4:** HAMD, MS, HAMA, and PA score and the reduction rate at week 2 after primary treatment.

	Group	Mean ± SD	Reduction rate (% ± SD)^a^	*F*/*H*	*p* ^b^
HAMD‐17	OFC (*n* = 20)	14.30 ± 5.85	35.43 ± 23.91	2.120	0.130
	DLPFC (*n* = 18)	10.33 ± 5.02	45.96 ± 27.22		
	Sham (*n* = 19)	10.26 ± 5.91	51.91 ± 24.98		
MS	OFC (*n* = 20)	6.90 ± 3.35	36.27 ± 30.95	1.010	0.371
	DLPFC (*n* = 18)	5.28 ± 2.65	49.07 ± 26.57		
	Sham (*n* = 19)	5.37 ± 3.47	48.40 ± 36.33		
HAMA	OFC (*n* = 20)	8.90 ± 5.21	46.37 ± 28.37	0.336	0.845
	DLPFC (*n* = 18)	8.67 ± 5.67	36.55 ± 45.49		
	Sham (*n* = 19)	7.84 ± 5.56	43.32 ± 41.78		
PA	OFC (*n* = 20)	6.45 ± 3.43	40.48 ± 30.96	0.025	0.976
	DLPFC (*n* = 18)	6.06 ± 3.83	38.52 ± 37.81		
	Sham (*n* = 19)	6.11 ± 3.83	40.99 ± 37.72		

Abbreviations: DLPFC, dorsolateral prefrontal cortex; HAMA, the Hamilton Anxiety Rating Scale; HAMD‐17, the 17‐item Hamilton Depression Rating Scale; MS, Maier subscale; OFC, orbitofrontal cortex; PA, psychic anxiety.

^a^ Reduction rate, calculated as (score at baseline—score at week 2)/score at baseline) × 100.

^b^ *p* values of one‐way ANOVA (HAMD‐17, MS, and PA) or Kruskal–Wallis test (HAMA).

The difference in the reduction rates among the three groups was statistically significant after maintenance treatment at week 4 (HAMD‐17: *H* (df = 2) = 12.511, *p* = 0.002; MS: *F*(2,49) = 4.738, *p* = 0.013). Post hoc multiple comparisons showed that the reduction rate of HAMD‐17 score in the OFC group was lower than that in the Sham group (adjusted *p* = 0.009) and the DLPFC group (adjusted *p* = 0.006). And the reduction rate of MS score in the OFC group was only significantly lower than that in the Sham group (adjusted *p* = 0.022) (Table 5).

**TABLE 5:** HAMD, MS, HAMA, and PA score and the reduction rate at week 4 after maintenance treatment.

	Group	Mean ± SD	Reduction rate (% ± SD)^a^	*F*/*H*	*p* ^b^
HAMD‐17	OFC (*n* = 19)	13.53 ± 5.18	39.10 ± 22.21	12.511	0.002**
	DLPFC (*n* = 17)	7.00 ± 3.95	62.38 ± 24.83		
	Sham (*n* = 16)	7.56 ± 4.38	63.97 ± 19.93		
MS	OFC (*n* = 19)	6.68 ± 2.79	38.59 ± 27.12	4.738	0.013*
	DLPFC (*n* = 17)	3.88 ± 2.32	61.17 ± 26.25		
	Sham (*n* = 16)	3.63 ± 2.80	65.04 ± 30.18		
HAMA	OFC (*n* = 19)	11.68 ± 6.34	29.82 ± 31.81	2.677	0.079
	DLPFC (*n* = 17)	6.65 ± 4.36	50.11 ± 27.53		
	Sham (*n* = 16)	6.00 ± 3.76	52.02 ± 36.13		
PA	OFC (*n* = 19)	7.84 ± 4.00	29.08 ± 32.81	2.613	0.084
	DLPFC (*n* = 17)	4.82 ± 2.79	49.58 ± 23.77		
	Sham (*n* = 16)	5.06 ± 2.67	48.36 ± 33.19		

Post hoc test				
	HAMD‐17	Adjusted *p* ^c^	MS	Adjusted *p* ^c^
OFC vs. Sham		0.009**		0.022*
DLPFC vs. Sham		1.000		1.000
OFC vs. DLPFC		0.006**		0.056

Abbreviations: DLPFC, dorsolateral prefrontal cortex; HAMA, the Hamilton Anxiety Rating Scale; HAMD‐17, the 17‐item Hamilton Depression Rating Scale; MS, Maier subscale; OFC, orbitofrontal cortex; PA, psychic anxiety.

^a^ Reduction rate, calculated as (score at baseline—score at week 4)/score at baseline) × 100.

^b^ *p* values of one‐way ANOVA (HAMA, MS, and PA) or Kruskal–Wallis test (HAMD‐17).

^c^ Adjusted *p* values of Bonferroni post hoc test of the reduction rate.

*p < 0.05; **p < 0.01.

All three groups exhibited a decrease in anxiety, as measured by HAMA and PA, although there was no significant difference in the reduction rates among the three groups at week 2 (HAMA: *H* (df = 2) = 0.336, *p* = 0.845; PA: *F*(2,54) = 0.025, *p* = 0.976) (Table 4) and week 4 (HAMS: *F*(2,49) = 2.677, *p* = 0.079; PA: *F*(2,49) = 2.613, *p* = 0.084) (Table 5).

The reduction rate of depression and anxiety scale score is shown in Figure 1.

### 3.4 Assessment of 6‐ and 8‐Week Follow‐Ups

At the 6‐ and 8‐week follow‐ups (Table S2), DSSS, SS, and SA scores were significant on the session's main effect, suggesting that scores reduced significantly from baseline to each time point. The group's main effect was significant on the 6‐week DSSS score (*p* = 0.035) and the 6‐week (*p* = 0.019) and 8‐week (*p* = 0.011) SS scores. None of the session × group interaction effects for DSSS and SS scores were significant, while SA scores showed significance (*p* = 0.013) on the 6‐week session × group interaction effect, suggesting the change in SA scores varied across the three groups over time progressed (Figure S2C). Post hoc analysis suggested that both DSSS (*p* = 0.035) and SS (*p* = 0.017) scores showed a greater decrease in the DLPFC group than in the OFC group at week 6 but were not significantly different from the Sham group (Figure S2A,B). At week 8, there was a lower decrease in SS scores in the OFC group compared to the DLPFC group (*p* = 0.020).

For HAMD‐17 scores at 6‐ and 8‐week follow‐up (Table S2), significant differences were observed for the session main effects at week 6 (*p* < 0.001) and week 8 (*p* < 0.001), as well as for the group main effect at week 6 (*p* = 0.009), whereas no significant differences were observed for the session × group interaction effect. Post hoc analysis of the main effect of the group at week 6 showed that compared to OFC group, the DLPFC group (*p* = 0.011) showed more decrease in HAMD‐17 scale scores (Figure S2D).

In addition to significance on the session's main effect, HAMA (*p* = 0.044) showed significance on the 6‐week session × group interaction effect, indicating that by week 6, the magnitude of change in HAMA scores differed among the three groups as time progressed and the DLPFC group showed a better improvement than the OFC group (Figure S2E). No group's main effect showed significant difference (Table S2).

### 3.5 Predictive Factor Analysis

Analysis of clinical and demographic factors affecting depressive and somatic symptoms (Table S3) showed that the reduction rate of DSSS score (after 10 Interventions) was negatively associated with gender as female, SSI score, DSSS score, and SS score at baseline, regardless of the group. It also revealed a significant interaction (*p* = 0.021) between age and tDCS group, and further analysis showed that in the OFC group, older participants showed a higher DSSS score reduction rate. The significant interaction (*p* = 0.011) between single or not and group showed that participants in a relationship performed better than the single individuals in the DLPFC group.

Correlation analysis of clinical demographic data with improvement in anxiety symptoms found a positive correlation between age and the rate of HAMA score reduction (after 10 Interventions) (Table S4). Also, general linear model results showed that the higher the HAMA score at baseline, the higher the rate of HAMA score reduction after 10 interventions (*p* = 0.032), regardless of group.

### 3.6 Safety Outcomes

As shown in Table S5, all the adverse effects item ratings showed no differences among the three groups. Meanwhile, no patients had manic episodes during the intervention and follow‐up, with YMRS scores of ≤ 5.

## 4 Discussion

This double‐blind, randomized, sham‐controlled clinical study compared the efficacy of tDCS targeting DLPFC and OFC on somatic symptoms in patients with MDD. After 2 weeks of primary treatment, the DLPFC group demonstrated a more pronounced improvement in depressive somatic symptoms compared to the Sham group. At week 4 and follow‐up periods, the DLPFC group outperformed the Sham and OFC groups, but did not show a significant difference with the Sham group. No significant improvement in depression and anxiety symptoms was observed in the active groups compared to the Sham group. The tDCS interventions were well tolerated, with no major adverse events reported.

### 4.1 Effect on Somatic Symptoms

The research discovered that anodic stimulation of the left DLPFC demonstrated superior effectiveness in alleviating depressive somatic symptoms, whereas cathodic stimulation of the right OFC appeared ineffectual. There was no difference in improvement of core depressive symptoms among the three groups in terms of MS score, which excludes somatic symptoms, further confirming the efficacy of DLPFC in treating somatic symptoms in MDD. This implies the possibility of alleviating depressive somatic symptoms through activation of the left DLPFC. The DLPFC is a cognitive‐executive‐control brain region processing expectancy where cognitive modulations of pain may initiate, then trigger the descending pain modulation system and reward system to diminish or intensify one's pain experience depending on context [52]. Given the paucity of studies exploring the therapeutic influence of tDCS on somatic symptoms in depressive patients, this study's findings align with previous research on pain and sleep disorders, suggesting that anodic stimulation of the left DLPFC via tDCS might present a potent approach to ameliorate depressive somatic symptoms [36, 37].

In our research, it was observed that tDCS targeting OFC did not exhibit superior effectiveness compared to the Sham group. Neuroimaging research indicated a correlation between chronic pain and sustained hypofunction in OFC [53]. Moreover, an interesting correlation was discovered that the stronger the connectivity of the OFC, the more pronounced the reduction in pain intensity [39]. Therefore, anodal stimulation of the OFC to enhance its activity without cathodic stimulation of right OFC may contribute to alleviating somatic symptoms. Previous studies also found that anodal stimulation targeting the primary motor cortex can alleviate fatigue and pain, implying that the primary motor cortex could be another effective target for alleviating somatic symptoms in patients with MDD [54, 55].

Post‐treatment follow‐up revealed that tDCS sustained its impact on depressive somatic symptoms for up to a month, although with no significant efficacy difference between the DLPFC group and the sham group, which is consistent with the prolonged effect on depressive symptoms [26]. Intensified electrical stimulation, including a stronger current and twice‐daily sessions, has been shown to be more effective in other psychiatric disorders [56, 57]. Intensified stimulation may also have a stronger effect in patients with MDD. Further research with a larger sample size is imperative to better interpret the effects on depressive somatic symptoms of tDCS targeting different regions and underlying mechanisms.

Concerning somatic anxiety, neither the DLPFC nor the OFC groups showed any significant effect. However, at the 6‐week follow‐up, the DLPFC group appeared to outperform the OFC group, although this does not constitute definitive proof of its efficacy. While DLPFC and OFC have been linked to somatic anxiety, clinical research in this area remains scant. Only a single case report exists to support the effect of tDCS on somatic anxiety [40]. Thus, which brain region tDCS is effective in relieving somatic anxiety by stimulating is still unknown. Further exploration is required to identify a suitable tDCS treatment paradigm.

### 4.2 Efficacy on Depression Severity

Neither OFC‐tDCS nor DLPFC‐tDCS demonstrated superiority over the Sham group in terms of depressive symptoms. In fact, the OFC group's performance was inferior to that of the DLPFC group and Sham group in terms of HAMD‐17 scores. Several factors may elucidate the finding. First, the optimal electrode positioning for OFC‐tDCS in depression treatment remains undetermined, which may have influenced the results of this study. In light of prior research indicating that inhibitory stimulation of the right OFC (FP2) could potentially alleviate depression symptoms, the decision was made to place the cathode over the OFC (Fp2). Nevertheless, these findings were derived from self‐reported measures and lacked clinician evaluations and sham controls, and they were not conducted within a depression cohort [32, 33].

Second, a recent TMS‐EEG analysis identified that OFC activity in depressed patients was lower than that in healthy controls prior to treatment. However, following active rTMS treatment, OFC activity significantly increased in depressed patients. This suggests that activating the OFC could potentially mitigate depression symptoms. Contrarily, our study inhibited the right‐sided OFC, which may have diminished the antidepressant effect of tDCS [58]. In addition, similar to our study, a previous study found no difference in antidepressant efficacy between tDCS anodic stimulation of DLPFC and the Sham group, which assumed that low currents delivered under the Sham tDCS condition were biologically active and could not be discounted [23].

Moreover, medications taken during tDCS treatment may also have an impact on the effectiveness of the treatment [59]. Medications that affect different neurotransmitter systems such as GABA, dopamine and 5‐hydroxytryptophan can all affect the effect of tDCS on tissue excitability [60]. Finally, our pilot study may have been insufficient in detecting differences. A study with a larger sample size could potentially uncover more pronounced differences and expanding the scope of the study would contribute to a more comprehensive understanding of these findings. It may be possible to improve the efficacy of tDCS in the treatment of depression by optimizing the therapeutic target, refining the current intensity, and controlling the clinical characteristics of the participants (e.g., minimizing the use of antidepressants), among other things.

### 4.3 Other Results

Our study did not find a significant improvement of tDCS treating anxiety symptoms of patients with MDD. A review of the effects of tDCS on anxiety in different samples showed some positive effects, but differences in the design and measures used did not provide conclusive evidence of its effectiveness for specific anxiety disorders or anxiety in depression [61]. The focus of this study was not on anxiety symptoms, but it is also worth further exploration.

The study assessed the predictive value of age and relationship status in response to tDCS intervention. Findings indicated that older participants in the OFC group and participants in a relationship in the DLPFC group experienced a more pronounced alleviation of depression and somatic symptoms. Furthermore, more severe baseline anxiety severity corresponded to a greater rate of HAMA score reduction. However, given the limited sample size of this preliminary study, it is necessary to conduct curative effect prediction research in a larger population in the future.

### 4.4 Limitations

There are several limitations that need to be mentioned in this study. Firstly, the restricted sample size and the predominance of online follow‐ups, a measure necessitated by the COVID‐19 pandemic, may have influenced the results. Secondly, some participants concurrently received antidepressants during the tDCS intervention, which is more practical in a clinical setting. The combined impact of tDCS and medication could potentially alter treatment efficacy [59, 60]. However, participants were required to maintain a consistent medication regimen for a minimum of 2 weeks before and throughout the 10 interventions. Moreover, further analysis revealed no significant correlation between medication use and effectiveness. Therefore, this factor is unlikely to have significantly affected the study outcomes. Finally, this study is a preliminary study focusing on somatic symptoms in patients with MDD. In further studies, parallel controlled trials should be carried out, combined with physiological measurement and cognitive evaluation, to investigate the efficacy of different targets of tDCS in MDD patients with different severity of somatic symptoms.

## 5 Conclusion

In conclusion, the tDCS treatment targeting DLPFC proved to be more effective in ameliorating depressive somatic symptoms than the sham procedure and appeared to surpass the stimulation of OFC. No significant improvement in the severity of anxiety and depression was observed in active groups compared to the sham group. Our study suggested the tDCS targeting DLPFC may be a potentially effective therapeutic target for alleviating somatic symptoms in patients with MDD.

## Conflicts of Interest

The authors declare no conflicts of interest.

## Supporting information


Appendix S1.

Appendix S2.

## Data Availability

The data that support the findings of this study are available on request from the corresponding author. The data are not publicly available due to privacy or ethical restrictions.

## References

[1] WHO . “WHO Depression,” 2023, updated March 31, 2023, https://www.who.int/en/news‐room/fact‐sheets/detail/depression.

[2] Collaborators GDaI , “Global Burden of 369 Diseases and Injuries in 204 Countries and Territories, 1990–2019: A Systematic Analysis for the Global Burden of Disease Study 2019,” Lancet 396, no. 10258 (2020): 1204–1222. 33069326 10.1016/S0140-6736(20)30925-9 PMC7567026

[3] B. W. Dunlop , S. Still , D. LoParo , et al., “Somatic Symptoms in Treatment‐Naive Hispanic and Non‐Hispanic Patients With Major Depression,” Depression and Anxiety 37, no. 2 (2020): 156–165. 31830355 10.1002/da.22984

[4] D. Novick , W. Montgomery , J. Aguado , et al., “Which Somatic Symptoms Are Associated With an Unfavorable Course in Asian Patients With Major Depressive Disorder?,” Journal of Affective Disorders 149, no. 1–3 (2013): 182–188. 23521872 10.1016/j.jad.2013.01.020

[5] M. Liu , “Potential Risk Factors of Cardiovascular Diseases and Mental Disorders,” Heart and Mind 7, no. 2 (2023): 55–56.

[6] G. I. Papakostas , P. McGrath , J. Stewart , et al., “Psychic and Somatic Anxiety Symptoms as Predictors of Response to Fluoxetine in Major Depressive Disorder,” Psychiatry Research 161, no. 1 (2008): 116–120. 18755514 10.1016/j.psychres.2008.02.011

[7] G. E. Simon , M. VonKorff , M. Piccinelli , C. Fullerton , and J. Ormel , “An International Study of the relation between somatic symptoms and depression,” New England Journal of Medicine 341 (1999): 1329–1335. 10536124 10.1056/NEJM199910283411801

[8] S. K. Chaturvedi and G. Desai , “Measurement and Assessment of Somatic Symptoms,” International Review of Psychiatry 25, no. 1 (2013): 31–40. 23383665 10.3109/09540261.2012.727787

[9] A. Tylee and P. Gandhi , “The Importance of Somatic Symptoms in Depression in Primary Care,” Prim Care Companion J Clin Psychiatry 07, no. 4 (2005): 167–176. 10.4088/pcc.v07n0405 PMC1192435 16163400

[10] R. F. Munoz , P. Cuijpers , F. Smit , A. Z. Barrera , and Y. Leykin , “Prevention of Major Depression,” Annual Review of Clinical Psychology 6 (2010): 181–212. 10.1146/annurev-clinpsy-033109-132040 20192789

[11] E. X. Lau and R. M. Rapee , “Prevention of Anxiety Disorders,” Current Psychiatry Reports 13, no. 4 (2011): 258–266. 21484451 10.1007/s11920-011-0199-x

[12] R. A. Munoz , M. E. McBride , A. J. Brnabic , et al., “Major Depressive Disorder in Latin America: The Relationship Between Depression Severity, Painful Somatic Symptoms, and Quality of Life,” Journal of Affective Disorders 86, no. 1 (2005): 93–98. 15820276 10.1016/j.jad.2004.12.012

[13] A. Quante , F. Regen , F. Schindler , et al., “Quetiapine as Combination Treatment With Citalopram in Unipolar Depression With Prominent Somatic Symptoms: A Randomised, Double‐Blind, Placebo‐Controlled Pilot Study,” Psychiatria Danubina 25, no. 3 (2013): 214–220. 24048387

[14] J. A. Yesavage , J. K. Fairchild , Z. Mi , et al., “Effect of Repetitive Transcranial Magnetic Stimulation on Treatment‐Resistant Major Depression in US Veterans,” JAMA Psychiatry 75, no. 9 (2018): 884. 29955803 10.1001/jamapsychiatry.2018.1483 PMC6142912

[15] A. R. Brunoni , A. H. Moffa , B. Sampaio‐Junior , et al., “Trial of Electrical Direct‐Current Therapy Versus Escitalopram for Depression,” New England Journal of Medicine 376, no. 26 (2017): 2523–2533. 28657871 10.1056/NEJMoa1612999

[16] R. Polania , M. A. Nitsche , and C. C. Ruff , “Studying and Modifying Brain Function With Non‐Invasive Brain Stimulation,” Nature Neuroscience 21, no. 2 (2018): 174–187. 29311747 10.1038/s41593-017-0054-4

[17] A. Antal , I. Alekseichuk , M. Bikson , et al., “Low Intensity Transcranial Electric Stimulation: Safety, Ethical, Legal Regulatory and Application Guidelines,” Clinical Neurophysiology 128, no. 9 (2017): 1774–1809. 28709880 10.1016/j.clinph.2017.06.001 PMC5985830

[18] A. R. Brunoni , M. A. Nitsche , N. Bolognini , et al., “Clinical Research With Transcranial Direct Current Stimulation (tDCS): Challenges and Future Directions,” Brain Stimulation 5, no. 3 (2012): 175–195. 22037126 10.1016/j.brs.2011.03.002 PMC3270156

[19] M. A. Nitsche and W. Paulus , “Excitability Changes Induced in the Human Motor Cortex by Weak Transcranial Direct Current Stimulation,” Journal of Physiology 527, no. Pt 3 (2000): 633–639. 10990547 10.1111/j.1469-7793.2000.t01-1-00633.x PMC2270099

[20] L. B. Razza , P. Palumbo , A. H. Moffa , et al., “A Systematic Review and Meta‐Analysis on the Effects of Transcranial Direct Current Stimulation in Depressive Episodes,” Depression and Anxiety 37, no. 7 (2020): 594–608. 32101631 10.1002/da.23004

[21] A. H. Moffa , D. Martin , A. Alonzo , et al., “Efficacy and Acceptability of Transcranial Direct Current Stimulation (tDCS) for Major Depressive Disorder: An Individual Patient Data Meta‐Analysis,” Progress in Neuro‐Psychopharmacology & Biological Psychiatry 99 (2020): 109836. 31837388 10.1016/j.pnpbp.2019.109836

[22] J. Hyde , H. Carr , N. Kelley , et al., “Efficacy of Neurostimulation Across Mental Disorders: Systematic Review and Meta‐Analysis of 208 Randomized Controlled Trials,” Molecular Psychiatry 27, no. 6 (2022): 2709–2719. 35365806 10.1038/s41380-022-01524-8 PMC8973679

[23] C. K. Loo , M. M. Husain , W. M. McDonald , et al., “International Randomized‐Controlled Trial of Transcranial Direct Current Stimulation in Depression,” Brain Stimulation 11, no. 1 (2018): 125–133. 29111077 10.1016/j.brs.2017.10.011

[24] F. Fregni , M. M. El‐Hagrassy , K. Pacheco‐Barrios , et al., “Evidence‐Based Guidelines and Secondary Meta‐Analysis for the Use of Transcranial Direct Current Stimulation in Neurological and Psychiatric Disorders,” International Journal of Neuropsychopharmacology 24, no. 4 (2021): 256–313. 32710772 10.1093/ijnp/pyaa051 PMC8059493

[25] A. V. Sathappan , B. M. Luber , and S. H. Lisanby , “The Dynamic Duo: Combining Noninvasive Brain Stimulation With Cognitive Interventions,” Progress in Neuro‐Psychopharmacology & Biological Psychiatry 89 (2019): 347–360. 30312634 10.1016/j.pnpbp.2018.10.006

[26] L. B. Razza , S. De Smet , A. Moffa , P. Sudbrack‐Oliveira , M. A. Vanderhasselt , and A. R. Brunoni , “Follow‐Up Effects of Transcranial Direct Current Stimulation (tDCS) for the Major Depressive Episode: A Systematic Review and Meta‐Analysis,” Psychiatry Research 302 (2021): 114024. 34058716 10.1016/j.psychres.2021.114024

[27] G. Csifcsak , N. M. Boayue , O. Puonti , A. Thielscher , and M. Mittner , “Effects of Transcranial Direct Current Stimulation for Treating Depression: A Modeling Study,” Journal of Affective Disorders 234 (2018): 164–173. 29529550 10.1016/j.jad.2018.02.077

[28] M. A. Salehinejad , E. Ghanavai , R. Rostami , and V. Nejati , “Cognitive Control Dysfunction in Emotion Dysregulation and Psychopathology of Major Depression (MD): Evidence From Transcranial Brain Stimulation of the Dorsolateral Prefrontal Cortex (DLPFC),” Journal of Affective Disorders 210 (2017): 241–248. 28064113 10.1016/j.jad.2016.12.036

[29] S. Grimm , J. Beck , D. Schuepbach , et al., “Imbalance Between Left and Right Dorsolateral Prefrontal Cortex in Major Depression Is Linked to Negative Emotional Judgment: An fMRI Study in Severe Major Depressive Disorder,” Biological Psychiatry 63, no. 4 (2008): 369–376. 17888408 10.1016/j.biopsych.2007.05.033

[30] N. Cardoner , V. Soria , M. Gratacos , et al., “Val66Met BDNF Genotypes in Melancholic Depression: Effects on Brain Structure and Treatment Outcome,” Depression and Anxiety 30, no. 3 (2013): 225–233. 23165919 10.1002/da.22025

[31] P. Fettes , L. Schulze , and J. Downar , “Cortico‐Striatal‐Thalamic Loop Circuits of the Orbitofrontal Cortex: Promising Therapeutic Targets in Psychiatric Illness,” Frontiers in Systems Neuroscience 11 (2017): 25. 28496402 10.3389/fnsys.2017.00025 PMC5406748

[32] R. Tadayonnejad , A. C. Wilson , S. A. Chu , et al., “Use of Right Orbitofrontal Repetitive Transcranial Magnetic Stimulation (rTMS) Augmentation for Treatment‐Refractory Obsessive‐Compulsive Disorder With Comorbid Major Depressive Disorder,” Psychiatry Research 317 (2022): 114856. 36155277 10.1016/j.psychres.2022.114856

[33] E. M. Khedr , K. Elbeh , M. Saber , Z. Abdelrady , and A. Abdelwarith , “A Double Blind Randomized Clinical Trial of the Effectiveness of Low Frequency rTMS Over Right DLPFC or OFC for Treatment of Obsessive‐Compulsive Disorder,” Journal of Psychiatric Research 156 (2022): 122–131. 36244200 10.1016/j.jpsychires.2022.10.025

[34] K. Pacheco‐Barrios , A. Cardenas‐Rojas , A. Thibaut , et al., “Methods and Strategies of tDCS for the Treatment of Pain: Current Status and Future Directions,” Expert Review of Medical Devices 17, no. 9 (2020): 879–898. 32845195 10.1080/17434440.2020.1816168 PMC7674241

[35] X. Qi , T. Jia , C. Zhang , et al., “The Different Analgesic Effects of Alpha High‐Definition Transcranial Alternating Current Stimulation Over the Primary Sensorimotor Cortex and the Left Dorsolateral Prefrontal Cortex During Sustained Experimental Pain,” Brain Stimulation 17, no. 2 (2024): 416–418. 38548132 10.1016/j.brs.2024.03.019

[36] J. P. Lefaucheur , A. Antal , S. S. Ayache , et al., “Evidence‐Based Guidelines on the Therapeutic Use of Transcranial Direct Current Stimulation (tDCS),” Clinical Neurophysiology 128, no. 1 (2017): 56–92. 27866120 10.1016/j.clinph.2016.10.087

[37] A. Herrero Babiloni , A. Bellemare , G. Beetz , et al., “The Effects of Non‐Invasive Brain Stimulation on Sleep Disturbances Among Different Neurological and Neuropsychiatric Conditions: A Systematic Review,” Sleep Medicine Reviews 55 (2021): 101381. 32992227 10.1016/j.smrv.2020.101381

[38] F. Estevez‐Lopez , H. H. Kim , M. Lopez‐Vicente , et al., “Physical Symptoms and Brain Morphology: A Population Neuroimaging Study in 12,286 Pre‐Adolescents,” Translational Psychiatry 13, no. 1 (2023): 254. 37438345 10.1038/s41398-023-02528-w PMC10338487

[39] A. Yoshino , Y. Okamoto , G. Okada , et al., “Changes in Resting‐State Brain Networks After Cognitive‐Behavioral Therapy for Chronic Pain,” Psychological Medicine 48, no. 7 (2018): 1148–1156. 28893330 10.1017/S0033291717002598

[40] S. Machado , L. O. Sant'Ana , B. Travassos , and D. Monteiro , “Anodal Transcranial Direct Current Stimulation Reduces Competitive Anxiety and Modulates Heart Rate Variability in an eSports Player,” Clinical Practice and Epidemiology in Mental Health 18 (2022): e174501792209270. 37274860 10.2174/17450179-v18-e2209270 PMC10156019

[41] C. K. Loo , A. Alonzo , D. Martin , P. B. Mitchell , V. Galvez , and P. Sachdev , “Transcranial Direct Current Stimulation for Depression: 3‐Week, Randomised, Sham‐Controlled Trial,” British Journal of Psychiatry 200, no. 1 (2012): 52–59. 10.1192/bjp.bp.111.097634 22215866

[42] A. F. Dasilva , M. E. Mendonca , S. Zaghi , et al., “tDCS‐Induced Analgesia and Electrical Fields in Pain‐Related Neural Networks in Chronic Migraine,” Headache 52, no. 8 (2012): 1283–1295. 22512348 10.1111/j.1526-4610.2012.02141.x PMC4166674

[43] H. Jasper , “Report of the Committee on Methods of Clinical Examination in Electroencephalography,” Electroencephalography and Clinical Neurophysiology 10, no. 2 (1958): 370–375.

[44] A. R. Brunoni , L. Valiengo , A. Baccaro , et al., “The Sertraline vs. Electrical Current Therapy for Treating Depression Clinical Study: Results From a Factorial, Randomized, Controlled Trial,” JAMA Psychiatry 70, no. 4 (2013): 383–391. 23389323 10.1001/2013.jamapsychiatry.32

[45] M. Hamilton , “Development of a Rating Scale for Primary Depressive Illness,” British Journal of Social and Clinical Psychology 6, no. 4 (1967): 278–296. 6080235 10.1111/j.2044-8260.1967.tb00530.x

[46] J. R. Fann , C. H. Bombardier , J. S. Richards , et al., “Venlafaxine Extended‐Release for Depression Following Spinal Cord Injury: A Randomized Clinical Trial,” JAMA Psychiatry 72, no. 3 (2015): 247–258. 25607727 10.1001/jamapsychiatry.2014.2482

[47] C. I. Hung , L. J. Weng , Y. J. Su , and C. Y. Liu , “Depression and Somatic Symptoms Scale: A New Scale With Both Depression and Somatic Symptoms Emphasized,” Psychiatry and Clinical Neurosciences 60, no. 6 (2006): 700–708. 17109704 10.1111/j.1440-1819.2006.01585.x

[48] M. Fava , “Somatic Symptoms, Depression, and Antidepressant Treatment,” Journal of Clinical Psychiatry 63, no. 4 (2002): 305–307. 12000203 10.4088/jcp.v63n0406

[49] M. Hamilton , “The Assessment of Anxiety States by Rating,” British Journal of Medical Psychology 32, no. 1 (1959): 50–55. 13638508 10.1111/j.2044-8341.1959.tb00467.x

[50] A. R. Brunoni , J. Amadera , B. Berbel , M. S. Volz , B. G. Rizzerio , and F. Fregni , “A Systematic Review on Reporting and Assessment of Adverse Effects Associated With Transcranial Direct Current Stimulation,” International Journal of Neuropsychopharmacology 14, no. 8 (2011): 1133–1145. 21320389 10.1017/S1461145710001690

[51] R. C. Young , J. T. Biggs , V. E. Ziegler , and D. A. Meyer , “A Rating Scale for Mania: Reliability, Validity and Sensitivity,” British Journal of Psychiatry 133 (1978): 429–435. 10.1192/bjp.133.5.429 728692

[52] Y. Tu , G. Wilson , J. Camprodon , et al., “Manipulating Placebo Analgesia and Nocebo Hyperalgesia by Changing Brain Excitability,” Proceedings of the National Academy of Sciences of the United States of America 118, no. 19 (2021): e2101273118. 33941677 10.1073/pnas.2101273118 PMC8126770

[53] A. Fumal , S. Laureys , L. Di Clemente , et al., “Orbitofrontal Cortex Involvement in Chronic Analgesic‐Overuse Headache Evolving From Episodic Migraine,” Brain 129, no. Pt 2 (2006): 543–550. 16330505 10.1093/brain/awh691

[54] M. Mortezanejad , F. Ehsani , N. Masoudian , M. Zoghi , and S. Jaberzadeh , “Comparing the Effects of Multi‐Session Anodal Trans‐Cranial Direct Current Stimulation of Primary Motor and Dorsolateral Prefrontal Cortices on Fatigue and Quality of Life in Patients With Multiple Sclerosis: A Double‐Blind, Randomized, Sham‐Controlled Trial,” Clinical Rehabilitation 34, no. 8 (2020): 1103–1111. 32397748 10.1177/0269215520921506

[55] S. Giannoni‐Luza , K. Pacheco‐Barrios , A. Cardenas‐Rojas , et al., “Noninvasive Motor Cortex Stimulation Effects on Quantitative Sensory Testing in Healthy and Chronic Pain Subjects: A Systematic Review and Meta‐Analysis,” Pain 161, no. 9 (2020): 1955–1975. 32453135 10.1097/j.pain.0000000000001893 PMC7679288

[56] E. Jafari , J. Alizadehgoradel , F. Pourmohseni Koluri , et al., “Intensified Electrical Stimulation Targeting Lateral and Medial Prefrontal Cortices for the Treatment of Social Anxiety Disorder: A Randomized, Double‐Blind, Parallel‐Group, Dose‐Comparison Study,” Brain Stimulation 14, no. 4 (2021): 974–986. 34167918 10.1016/j.brs.2021.06.005

[57] J. Alizadehgoradel , B. Molaei , K. Barzegar Jalali , et al., “Targeting the Prefrontal‐Supplementary Motor Network in Obsessive‐Compulsive Disorder With Intensified Electrical Stimulation in Two Dosages: A Randomized, Controlled Trial,” Translational Psychiatry 14, no. 1 (2024): 78. 38316750 10.1038/s41398-024-02736-y PMC10844238

[58] S. Han , X. X. Li , S. Wei , et al., “Orbitofrontal Cortex‐Hippocampus Potentiation Mediates Relief for Depression: A Randomized Double‐Blind Trial and TMS‐EEG Study,” Cell Reports Medicine 4, no. 6 (2023): 101060. 37263267 10.1016/j.xcrm.2023.101060 PMC10313932

[59] A. R. Brunoni , R. Ferrucci , M. Bortolomasi , et al., “Interactions Between Transcranial Direct Current Stimulation (tDCS) and Pharmacological Interventions in the Major Depressive Episode: Findings From a Naturalistic Study,” European Psychiatry 28, no. 6 (2013): 356–361. 23182847 10.1016/j.eurpsy.2012.09.001

[60] M. E. McLaren , N. R. Nissim , and A. J. Woods , “The Effects of Medication Use in Transcranial Direct Current Stimulation: A Brief Review,” Brain Stimulation 11, no. 1 (2018): 52–58. 29066167 10.1016/j.brs.2017.10.006 PMC5729094

[61] D. J. Stein , L. Fernandes Medeiros , W. Caumo , and I. L. Torres , “Transcranial Direct Current Stimulation in Patients With Anxiety: Current Perspectives,” Neuropsychiatric Disease and Treatment 16 (2020): 161–169. 32021208 10.2147/NDT.S195840 PMC6969693

