# Supplementary material for: Effects of Transcranial Direct Current Stimulation Targeting Dorsolateral Prefrontal Cortex and Orbitofrontal Cortex on Somatic Symptoms in Patients With Major Depressive Disorder: A Randomized, Double‐Blind, Controlled Clinical Trial
Source: CNS Neurosci Ther. 2024 Nov 8;30(11):e70110. doi: 10.1111/cns.70110 (PMC11549028; doi:10.1111/cns.70110)
Supplement: Supplementary file 1 — Appendix S1. [file CNS-30-e70110-s001.doc]

**Supplementary**

**Table S1**

The number of patients followed up at each time point.

|  | OFC group | DLPFC group | Sham group | Total |
| --- | --- | --- | --- | --- |
| Baseline | 23 | 23 | 24 | 70 |
| Week 2 | 20 (87.0%) | 18 (78.26%) | 19 (79.2%) | 57 (81.4%) |
| Week 4 | 19 (82.6%) | 17 (73.9%) | 16 (66.7%) | 52 (74.3%) |
| Week 6 | 15 (65.2%) | 15 (65.2%) | 11 (45.8%) | 41 (58.6%) |
| Week 8 | 11 (47.8%) | 11 (47.8%) | 5 (20.8%) | 27 (38.6%) |

Abbreviations: OFC: orbitofrontal cortex; DLPFC: dorsolateral prefrontal cortex.

**Table S2**

Session, Group and Interaction effects of HAMD-17, DSSS, SS, HAMA and SA scores at 6-week and 8-week follow-up.

|  | | Session effect | | Group effect | | Interaction | |  |  |
| --- | --- | --- | --- | --- | --- | --- | --- | --- | --- |
|  | | F | *P* | F | *P* | F | *P* |  |  |
| DSSS | 6-week | 53.151 | <0.001*** | 3.673 | 0.035* | 1.711 | 0.130 |  |  |
|  | 8-week | 22.457 | <0.001*** | 2.099 | 0.145 | 1.213 | 0.314 |  |  |
| SS | 6-week | 18.089 | <0.001*** | 4.386 | 0.019* | 0.996 | 0.075 |  |  |
|  | 8-week | 7.452 | 0.001** | 5.526 | 0.011* | 7.452 | 0.430 |  |  |
| SA | 6-week | 5.044 | <0.001*** | 1.964 | 0.154 | 8.444 | 0.013* |  |  |
|  | 8-week | 53.033 | 0.005** | 0.859 | 0.436 | 1.884 | 0.087 |  |  |
| HAMD-17 | 6-week | 82.118 | <0.001*** | 5.361 | 0.009** | 2.041 | 0.066 |  |  |
|  | 8-week | 16.185 | <0.001*** | 1.497 | 0.244 | 1.708 | 0.123 |  |  |
| HAMA | 6-week | 30.646 | <0.001*** | 1.644 | 0.207 | 2.244 | 0.044* |  |  |
|  | 8-week | 12.655 | <0.001*** | 0.299 | 0.745 | 1.307 | 0.249 |  |  |

Group's main effect analysis in follow-up

|  | 6-week follow-up | | | 8-week follow-up | | |
| --- | --- | --- | --- | --- | --- | --- |
|  | Mean difference | SEM | Adjusted  *P* | Mean  difference | SEM | Adjusted  *P* |
| DSSS |  |  |  |  |  |  |
| OFC vs SHAM | 2.241 | 3.240 | 1.000 | 3.844 | 4.625 | 1.000 |
| DLPFC vs SHAM | -5.642 | 3.240 | 0.269 | -3.647 | 4.625 | 1.000 |
| OFC vs DLPFC | 7.883 | 2.980 | 0.035* | 7.491 | 3.657 | 0.155 |
| SS |  |  |  |  |  |  |
| OFC vs SHAM | 2.665 | 1.540 | 0.287 | 4.884 | 1.905 | 0.056 |
| DLPFC vs SHAM | -1.485 | 1.540 | 0.990 | 0.429 | 1.905 | 1.000 |
| OFC vs DLPFC | 4.150 | 1.416 | 0.017* | 4.455 | 1.506 | 0.020* |
| SA |  |  |  |  |  |  |
| OFC vs SHAM | 1.468 | 0.761 | 0.178 | 1.436 | 1.134 | 0.622 |
| DLPFC vs SHAM | 0.568 | 0.761 | 1.000 | 0.709 | 1.134 | 1.000 |
| OFC vs DLPFC | 0.900 | 0.700 | 0.603 | 0.727 | 0.897 | 1.000 |
| HAMD-17 |  |  |  |  |  |  |
| OFC vs SHAM | 3.550 | 1.502 | 0.070 | 2.116 | 2.424 | 1.000 |
| DLPFC vs SHAM | -0.733 | 1.502 | 1.000 | -1.175 | 2.424 | 1.000 |
| OFC vs DLPFC | 4.283 | 1.382 | 0.011* | 3.291 | 1.916 | 0.296 |
| HAMA |  |  |  |  |  |  |
| OFC vs SHAM | 2.495 | 1.606 | 0.386 | 1.582 | 2.586 | 1.000 |
| DLPFC vs SHAM | 0.212 | 1.606 | 1.000 | 0.200 | 2.586 | 1.000 |
| OFC vs DLPFC | 2.283 | 1.478 | 0.392 | 1.382 | 2.045 | 1.000 |

**P*＜0.05 ***P*＜0.01 ****P*＜0.001

Abbreviations: DSSS: Depression and Somatic Symptoms Scale; SS: somatic subscale; SA: somatic anxiety; HAMD-17: the 17-item Hamilton Depression Rating Scale; HAMA: the Hamilton Anxiety Rating Scale; OFC: orbitofrontal cortex; DLPFC: dorsolateral prefrontal cortex.

**Table S3**

Analysis of clinical and demographic factors influencing DSSS reduction rate (after 10 Interventions).

| Independent variable | | | Wald χ^2^ | | *P* | r |
| --- | --- | --- | --- | --- | --- | --- |
| Gender -  Female vs Male |  |  | |  | | -0.273* |
|  | Group main effect | | 8.973 | | 0.011* |  |
|  | Trait main effect | | 2.493 | | 0.114 |  |
|  | Interaction | | 0.492 | | 0.782 |  |
| Age |  |  | |  | | 0.127 |
|  | Group main effect | | 8.516 | | 0.014* |  |
|  | Trait main effect | | 0.391 | | 0.532 |  |
|  | Interaction | | 7.760 | | 0.021* |  |
| Education |  |  | |  | | 0.191 |
|  | Group main effect | | 1.634 | | 0.442 |  |
|  | Trait main effect | | 0.773 | | 0.379 |  |
|  | Interaction | | 2.564 | | 0.278 |  |
| Single –  Yes vs No |  |  | |  | | -0.054 |
|  | Group main effect | | 18.868 | | <0.001*** |  |
|  | Trait main effect | | 0.753 | | 0.386 |  |
|  | Interaction | | 9.099 | | 0.011* |  |
| Employment –  Yes vs No |  |  | |  | | 0.150 |
|  | Group main effect | | 4.558 | | 0.102 |  |
|  | Trait main effect | | 1.247 | | 0.264 |  |
|  | Interaction | | 0.992 | | 0.609 |  |
| Age at first onset |  |  | |  | | 0.161 |
|  | Group main effect | | 2.541 | | 0.281 |  |
|  | Trait main effect | | 0.906 | | 0.341 |  |
|  | Interaction | | 1.137 | | 0.566 |  |
| On medication –  Yes vs No |  |  | |  | | 0.126 |
|  | Group main effect | | 13.692 | | 0.001** |  |
|  | Trait main effect | | 0.781 | | 0.377 |  |
|  | Interaction | | 2.340 | | 0.310 |  |
| SSI score at baseline |  |  | |  | | -0.322* |
|  | Group main effect | | 7.279 | | 0.026* |  |
|  | Trait main effect | | 4.714 | | 0.030* |  |
|  | Interaction | | 0.780 | | 0.677 |  |
| DSSS score at baseline |  |  | |  | | -0.399** |
|  | Group main effect | | 8.641 | | 0.013* |  |
|  | Trait main effect | | 8.510 | | 0.004** |  |
|  | Interaction | | 4.174 | | 0.124 |  |
| SS score at baseline |  |  | |  | | -0.350** |
|  | Group main effect | | 16.557 | | <0.001*** |  |
|  | Trait main effect | | 13.571 | | <0.001*** |  |
|  | Interaction | | 6.002 | | 0.050 |  |

**P*＜0.05 ***P*＜0.01 ****P*＜0.001

Abbreviations: DSSS: Depression and Somatic Symptoms Scale; SS: somatic subscale; SSI: Beck Scale for Suicide Ideation.

**Table S4**

Analysis of clinical and demographic factors influencing HAMA reduction rate (after 10 Interventions).

| Independent variable | | | F | | *P* | r |
| --- | --- | --- | --- | --- | --- | --- |
| Gender -  Female vs Male |  |  | |  | | 0.066 |
|  | Group main effect | | 0.549 | | 0.581 |  |
|  | Trait main effect | | 0.419 | | 0.520 |  |
|  | Interaction | | 2.455 | | 0.096 |  |
| Age |  |  | |  | | 0.291* |
|  | Group main effect | | 0.512 | | 0.602 |  |
|  | Trait main effect | | 3.796 | | 0.057 |  |
|  | Interaction | | 0.524 | | 0.596 |  |
| Education |  |  | |  | | 0.099 |
|  | Group main effect | | 0.151 | | 0.860 |  |
|  | Trait main effect | | 0.323 | | 0.572 |  |
|  | Interaction | | 0.031 | | 0.970 |  |
| Single –  Yes vs No |  |  | |  | | -0.060 |
|  | Group main effect | | 3.544 | | 0.036* |  |
|  | Trait main effect | | 0.004 | | 0.949 |  |
|  | Interaction | | 0.722 | | 0.491 |  |
| Employment –  Yes vs No |  |  | |  | | 0.036 |
|  | Group main effect | | 1.721 | | 0.189 |  |
|  | Trait main effect | | 0.699 | | 0.407 |  |
|  | Interaction | | 0.619 | | 0.542 |  |
| Age at first onset |  |  | |  | | 0.109 |
|  | Group main effect | | 0.853 | | 0.432 |  |
|  | Trait main effect | | 1.210 | | 0.277 |  |
|  | Interaction | | 0.773 | | 0.467 |  |
| On medication –  Yes vs No |  |  | |  | | -0.161 |
|  | Group main effect | | 2.455 | | 0.096 |  |
|  | Trait main effect | | 0.344 | | 0.560 |  |
|  | Interaction | | 0.151 | | 0.860 |  |
| SSI score at baseline |  |  | |  | | -0.151 |
|  | Group main effect | | 1.847 | | 0.168 |  |
|  | Trait main effect | | 1.082 | | 0.303 |  |
|  | Interaction | | 0.776 | | 0.466 |  |
| HAMA score at baseline |  |  | |  | | 0.193 |
|  | Group main effect | | 0.796 | | 0.457 |  |
|  | Trait main effect | | 4.845 | | 0.032* |  |
|  | Interaction | | 1.644 | | 0.203 |  |
| SA score at baseline |  |  | |  | | 0.141 |
|  | Group main effect | | 0.401 | | 0.672 |  |
|  | Trait main effect | | 3.476 | | 0.068 |  |
|  | Interaction | | 1.110 | | 0.337 |  |

**P*＜0.05 ***P*＜0.01 ****P*＜0.001

Abbreviations: HAMD-17: the 17-item Hamilton Depression Rating Scale; SSI: Beck Scale for Suicide Ideation; HAMA: the Hamilton Anxiety Rating Scale; SA: somatic anxiety.

**Table S5**

Adverse effects severity among three groups.

| Adverse effect | Group | Severity, n | | Fisher’s exact | *P* |
| --- | --- | --- | --- | --- | --- |
|  |  | Mild | Moderate |  |  |
| Headache | OFC group |  | 3 | 4.586 | 0.229 |
|  | DLPFC group | 2 |  |  |  |
|  | Sham group | 2 | 1 |  |  |
| Scalp pain | OFC group | 2 | 1 | 1.968 | 1.000 |
|  | DLPFC group | 3 |  |  |  |
|  | Sham group | 1 |  |  |  |
| Itching | OFC group | 2 |  | 1.331 | 1.000 |
|  | DLPFC group | 4 | 1 |  |  |
|  | Sham group | 2 |  |  |  |
| Sleepiness | OFC group | 3 | 1 | 1.051 | 1.000 |
|  | DLPFC group | 2 |  |  |  |
|  | Sham group | 2 | 1 |  |  |

Abbreviations: OFC: orbitofrontal cortex; DLPFC: dorsolateral prefrontal cortex.

**Table S6**

Response and remission rates of HAMD-17 after 10 and 12 interventions.

|  | HAMD-17, n (%) | |
| --- | --- | --- |
|  | Response^a^ | Remission^b^ |
| After 10 interventions |  |  |
| OFC group (n=20) | 6 (30.0%) | 2 (10.0%) |
| DLPFC group (n=18) | 9 (50.0%) | 6 (33.3%) |
| Sham group (n=19) | 9 (47.4%) | 7 (36.8%) |
| χ2 | 1.878 | 4.289 |
| *P*^c^ | 0.391 | 0.117 |
| After 12 interventions |  |  |
| OFC group (n=19) | 6 (31.6%) | 2 (10.5%) |
| DLPFC group (n=17) | 14 (82.4%) | 10 (58.8%) |
| Sham group (n=16) | 14 (87.5%) | 8 (50.0%) |
| χ2 | 15.214 | 10.142 |
| *P*^c^ | 0.000*** | 0.006** |

^a^ Response: at least a 50% reduction in scales scores from baseline.

^b^ Remission for the HAMD 17 is 7 or less.

^c^ *P* values of Pearson’s chi-squared test.

***P*＜0.01 ****P* < 0.001

Abbreviations: HAMD-17: the 17-item Hamilton Depression Rating Scale; OFC: orbitofrontal cortex; DLPFC: dorsolateral prefrontal cortex.

**Table S7**

Reduction rates of DSSS, HAMD, and HAMA score in male and female groups at week 2 and week 4.

| Week 2 | Group | Male | Female | | |
| --- | --- | --- | --- | --- | --- |
|  |  | Reduction rate (%±SD)^a^ | Reduction rate (%±SD)^a^ | t/Z | *P*^b^ |
| DSSS | OFC group | 46.80±26.18 | 32.01±26.32 | 1.089 | 0.290 |
|  | DLPFC group | 62.88±25.34 | 47.37±26.81 | 1.177 | 0.256 |
|  | Sham group | 30.73±40.61 | 27.01±11.96 | 0.157 | 0.889 |
| HAMD-17 | OFC group | 47.28±30.21 | 31.49±21.17 | 1.302 | 0.209 |
|  | DLPFC group | 38.48±39.02 | 49.70±20.18 | -0.661 | 0.532 |
|  | Sham group | 71.91±13.40 | 48.17±25.10 | 1.571 | 0.135 |
| HAMA | OFC group | 61.49±34.15 | 41.33±25.49 | 1.412 | 0.175 |
|  | DLPFC group | 46.68±37.74 | 31.48±49.66 | -0.656 | 0.512 |
|  | Sham group | 62.47±30.65 | 39.73±43.39 | -0.894 | 0.371 |
| Week 4 |  | | | | |
| DSSS | OFC group | 66.53±27.05 | 39.81±25.20 | 1.860 | 0.080 |
|  | DLPFC group | 71.85±24.15 | 68.02±21.72 | 0.335 | 0.742 |
|  | Sham group | 54.63±62.51 | 42.64±34.77 | 0.469 | 0.646 |
| HAMD-17 | OFC group | 12.60±8.91 | 14.87±4.73 | -0.741 | 0.468 |
|  | DLPFC group | 10.00±5.83 | 10.50±4.83 | -0.194 | 0.849 |
|  | Sham group | 6.33±3.21 | 11.00±6.07 | -1.278 | 0.218 |
| HAMA | OFC group | 40.08±33.01 | 27.09±32.09 | 0.715 | 0.484 |
|  | DLPFC group | 39.28±39.07 | 56.02±18.45 | -0.991 | 0.359 |
|  | Sham group | 39.50±59.60 | 54.91±31.49 | -0.539 | 0.590 |

^a^ Reduction rate, calculated as (score at baseline - score at week 2/4) / score at baseline) × 100.

^b^ *P* values of Independent Samples t-test or Mann-Whitney U test (week 2: HAMA (DLPFC and Sham), week 4: HAMA (Sham)).

Abbreviations: DSSS: Depression and Somatic Symptoms Scale; HAMD-17: the 17-item Hamilton Depression Rating Scale; HAMA: the Hamilton Anxiety Rating Scale; OFC: orbitofrontal cortex; DLPFC: dorsolateral prefrontal cortex.

**
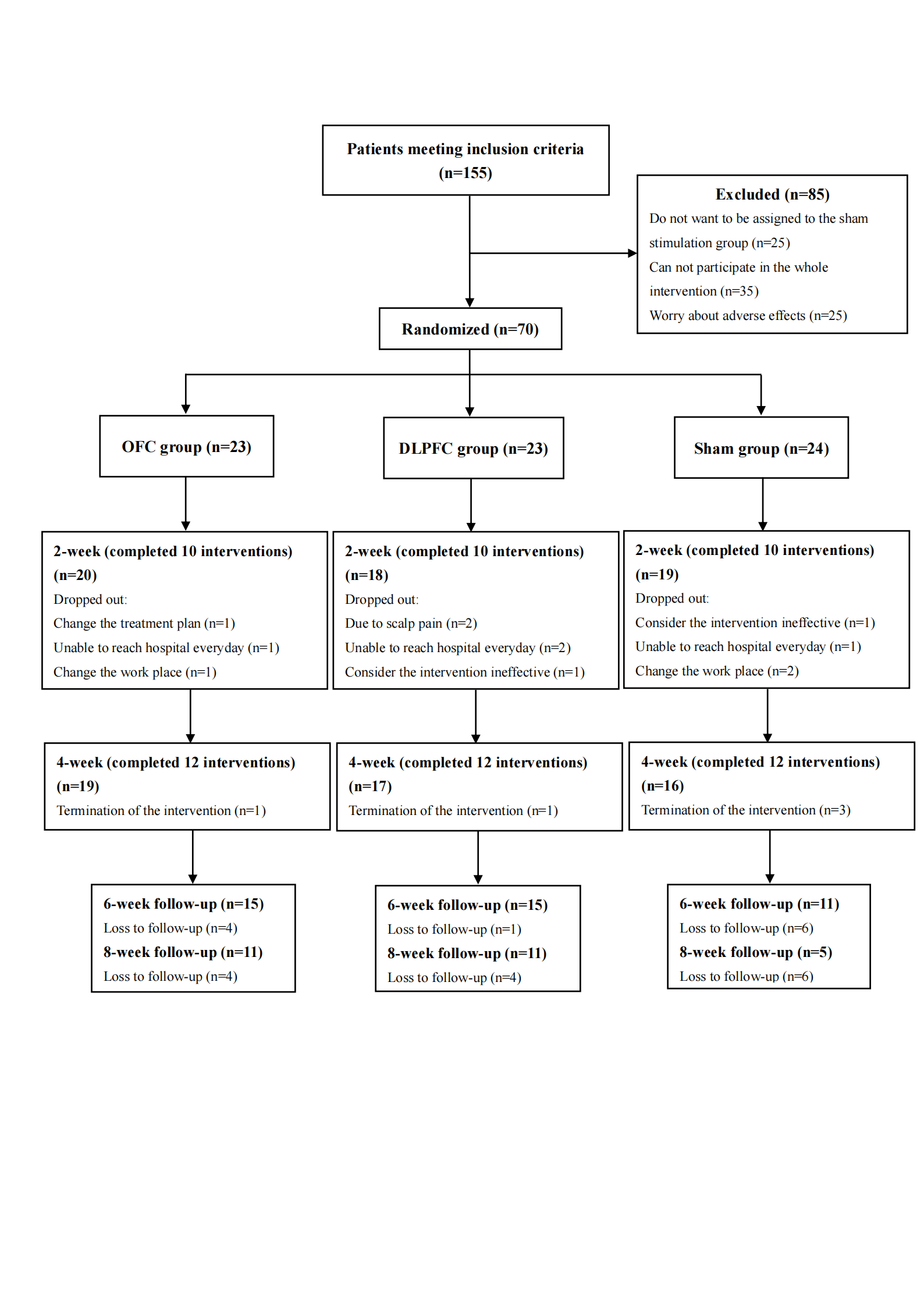
Fig. S1.** CONSORT flow chart of the study

**A**


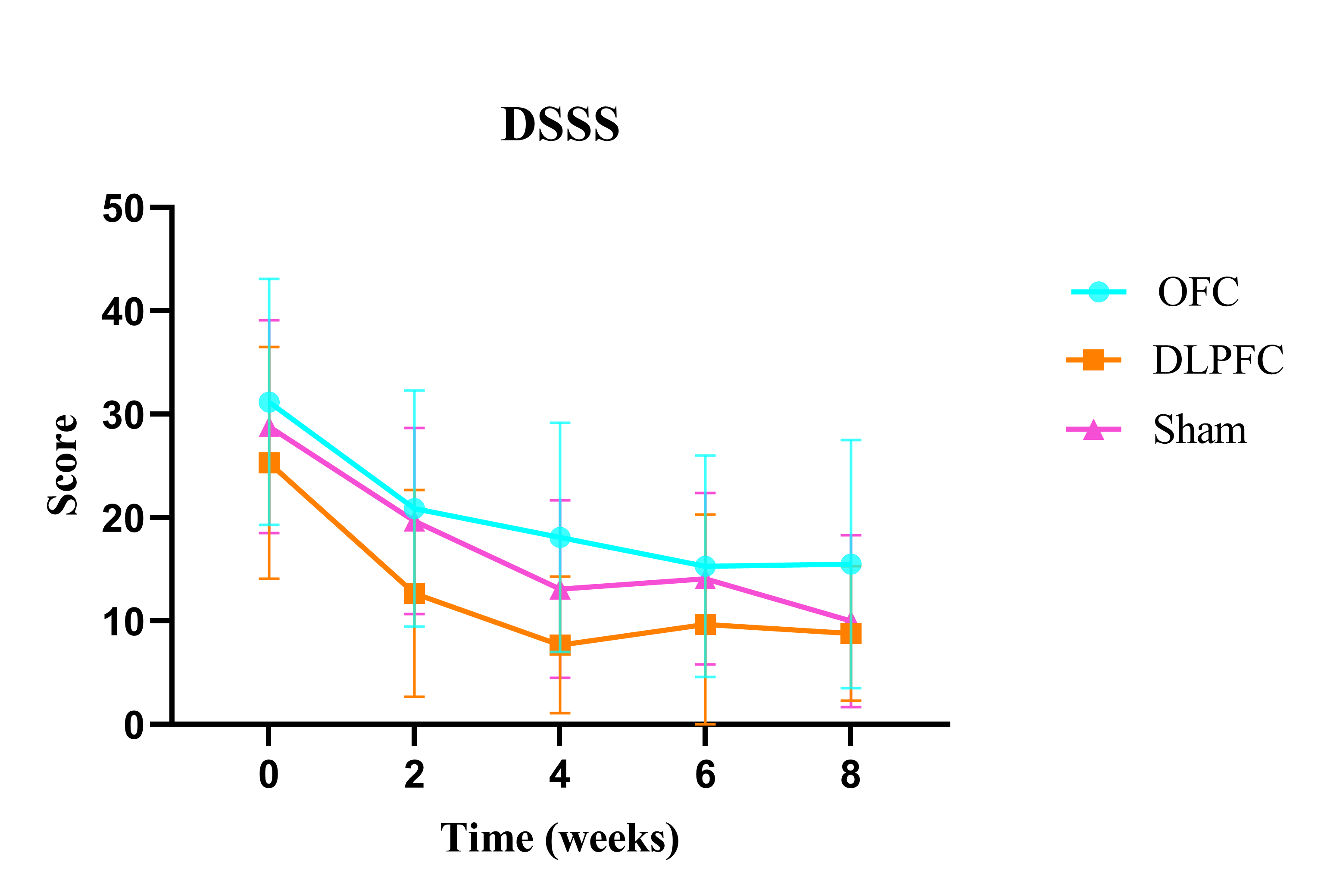


**B**


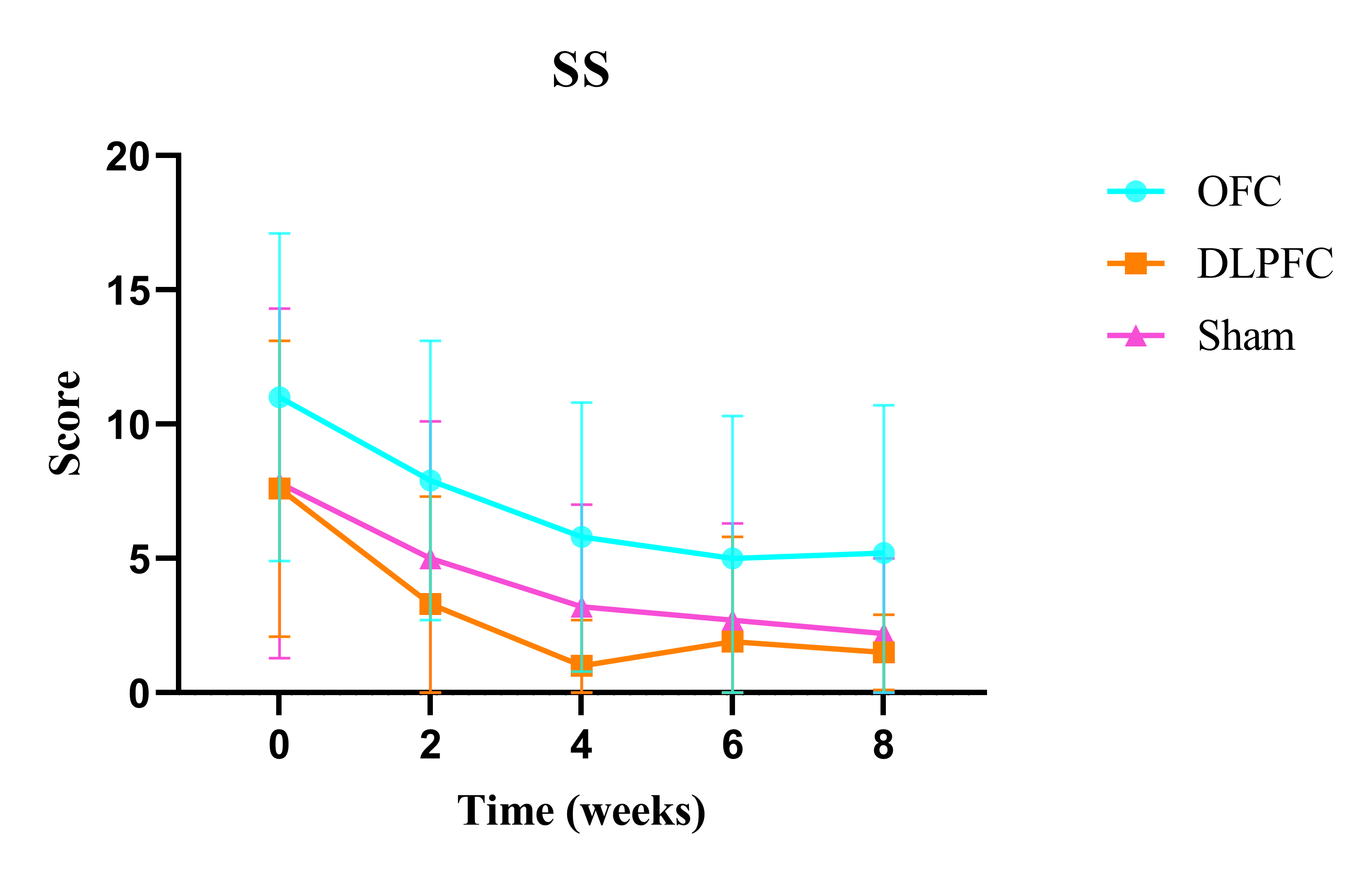


**C**

**
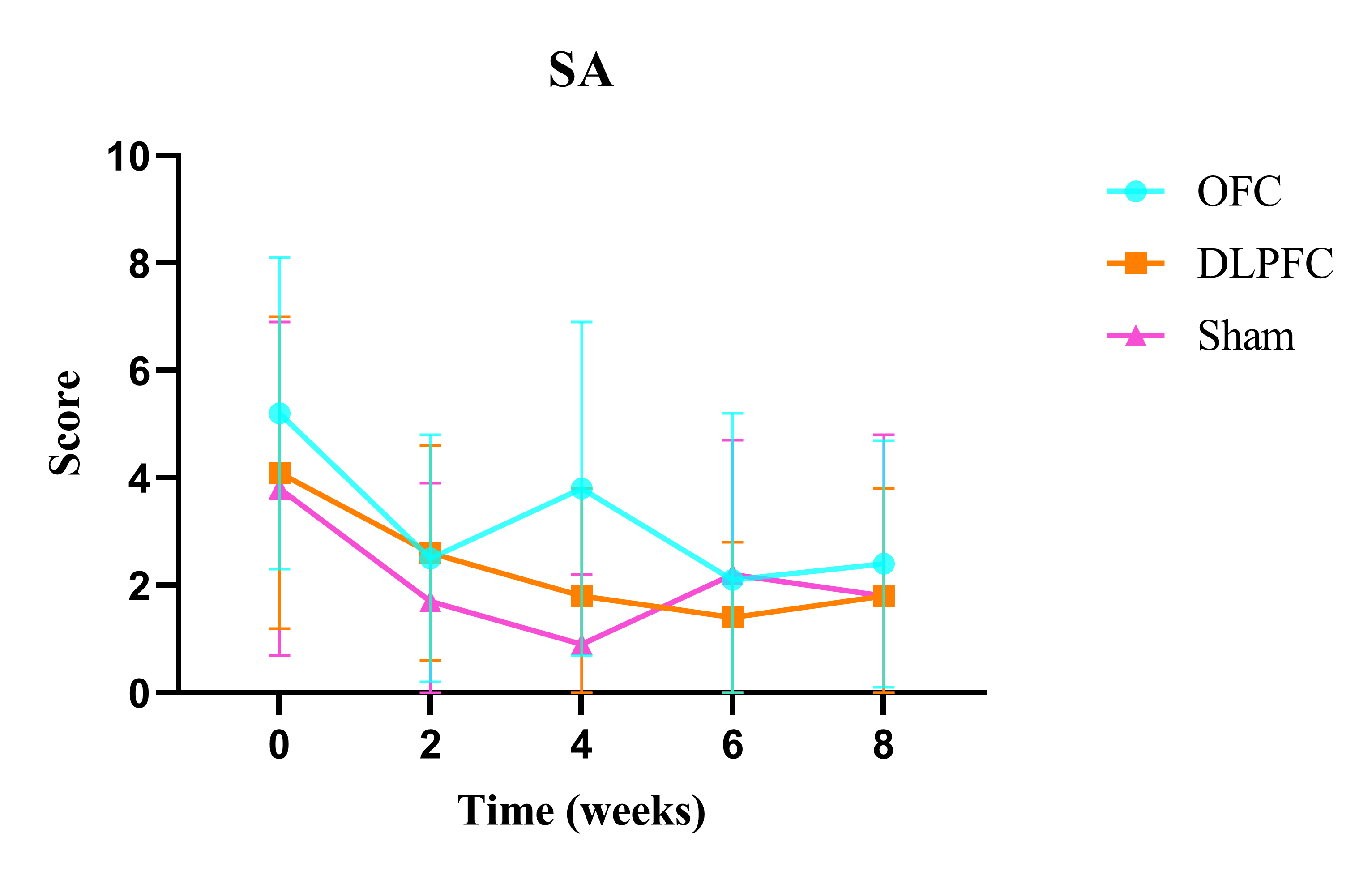
**

**D**


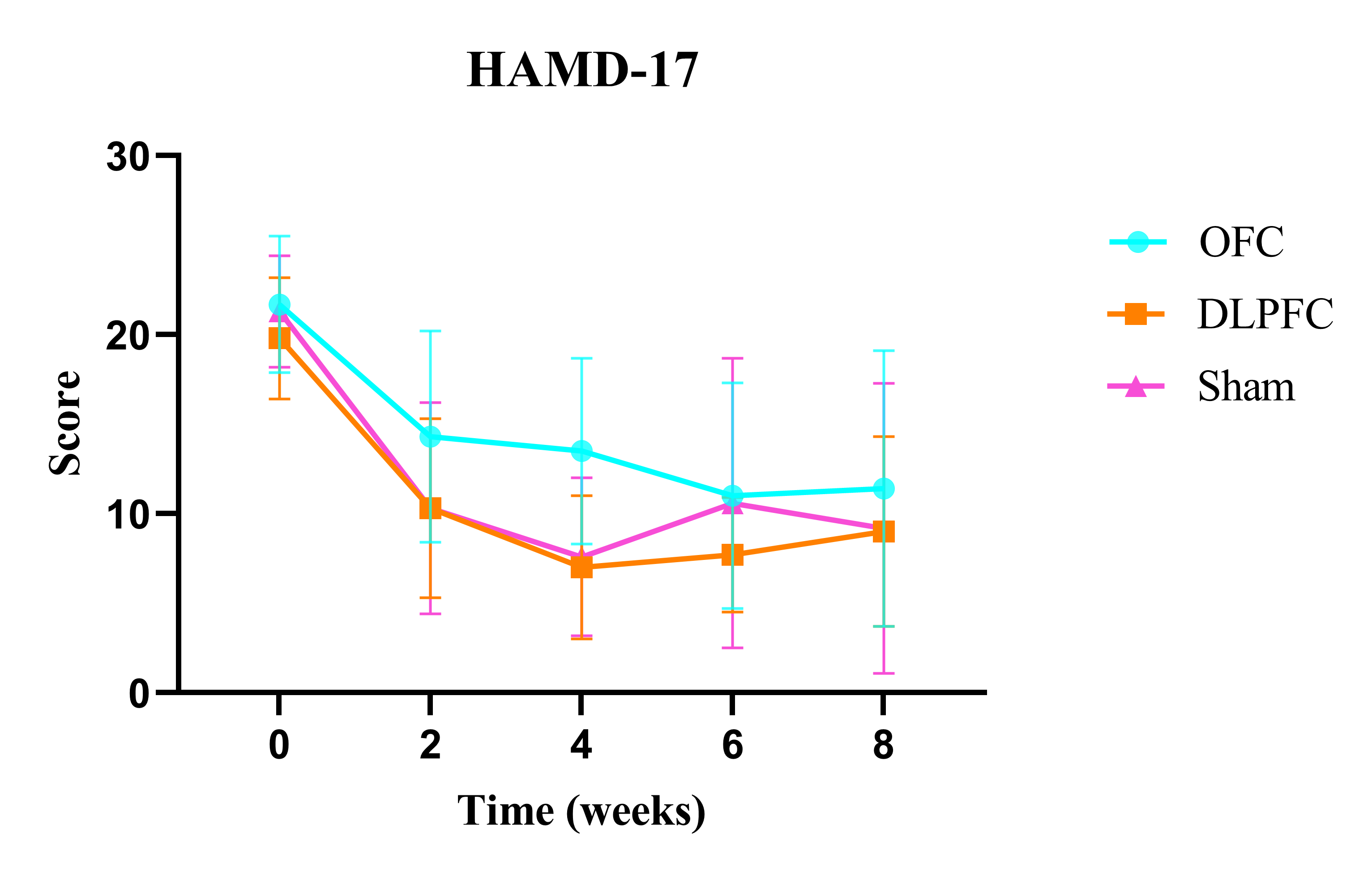


**E**


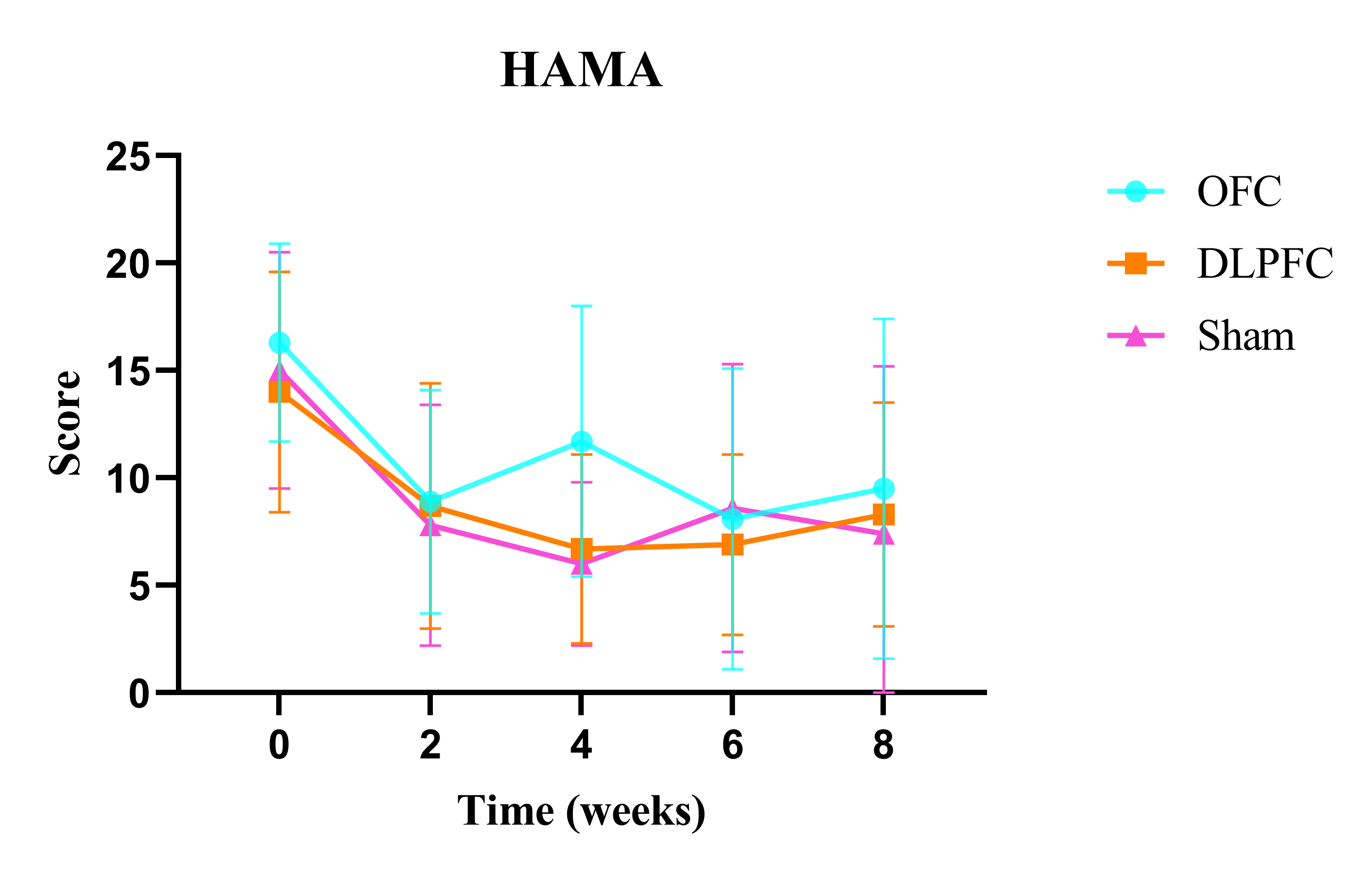


**Fig. S2.** DSSS, SS, SA, HAMD-17 and HAMA scores for three groups over intervention phases and follow-up (Mean ± SD). Abbreviations: DSSS: Depression and Somatic Symptoms Scale; SS: somatic subscale; SA: somatic anxiety; HAMD-17: the 17-item Hamilton Depression Rating Scale; HAMA: the Hamilton Anxiety Rating Scale; OFC: orbitofrontal cortex; DLPFC: dorsolateral prefrontal cortex.
